# Effectiveness of unilateral sequential video-assisted sympathetic chain blockage for primary palmar hyperidrosis in children and adolescents

**DOI:** 10.3389/fped.2022.1067141

**Published:** 2022-11-23

**Authors:** Ottavio Adorisio, Enrico Davoli, Emanuela Ceriati, Sonia Battaglia, Daniela Camanni, Francesco De Peppo

**Affiliations:** ^1^ Department of Pediatric Surgery, Pediatric Surgery Unit, Bambino Gesù Children’s Hospital, IRCCS, Rome, Italy; ^2^ Department of General Surgery, Campus Biomedico University Hospital, Rome, Italy

**Keywords:** palmar hyperhidrosis, sweating, thoracoscopy, sympathecotomy, compensatory hyperhidrosis

## Abstract

**Introduction:**

Primary palmar hyperhidrosis (PPH) is a severely debilitating condition that can affect patients of any age. Thoracoscopic sympathectomy provides a definitive treatment for PPH. Aim of this study is to investigate the effectiveness of unilateral sequential video-assisted thoracic sympathetic chain clamping (VATSCC) by clips application in pediatric population.

**Methods:**

The surgical procedure was done in the semi-sitting position, under general anesthesia with orotracheal intubation. Mean operation time was 23 ± 6 min (range 12–45). Two 5 mm ports were inserted at the level of the middle axillary line in the second and fourth intercostal space respectively. The sympathetic chain was identified, and two clips were applied, the first one at the level of the third and the second one, at the level of the fourth rib. No chest tube was used. Resolution of symptoms, complications, recurrence rate, onset and duration of compensatory hyperhidrosis were analyzed.

**Results:**

From August 2017 to September 2021, 58 patients (male:female ratio 32:26), mean age 16.5 years (range 14–19), with PPH underwent unilateral sequential VATSCC by clips application, starting on the dominant hand. The contralateral side was operated 2 months after. All patients except one (transient pneumothorax) were discharged on the first post-op day. No immediate or late complications have been recorded. Mean follow-up was 32 months (range 6–41). All patients except one (1,7%), affected by Raynaud's disease, showed a complete resolution of the symptom. Seven patients (12%) developed transient moderate compensative hyperhidrosis (CH) that spontaneously disappeared in the postoperative period.

**Conclusions:**

Unilateral sequential thoracoscopic sympathetic chain clamping for PPH in pediatric patients is a safe and very effective procedure with a low complication rate and low incidence of postoperative CH that, in our experience, resolved spontaneously in the postoperative period, after the second surgery leading to an improvement in the quality of life.

## Introduction

Primary palmar hyperhidrosis (PPH) is a somatic disorder causing excessive sweating of the hands, affecting approximately 1% of the general population (1). This feature usually starts during puberty but, in some cases, PPH can present in early childhood leading to bad smell, severe emotional and social handicaps with avoidance of social relationships, reflecting also on scholarly activities (2). The uncomfortable feelings and continuous wetting of the clothes may negatively affect the quality of life in a very significant way (3).

Video-assisted thoracoscopic sympathectomy (VATS) represents a viable option for the treatment of PPH. It is a safe procedure with good results (4), but often, compensatory hyperhidrosis (CH) may complicate the postoperative course. It has been reported that CH can occur in 3%–98% of patients after surgery, leading to increased sweating in other parts of the body such as the back, abdomen, or groin (5).

This study aims to evaluate the feasibility and effectiveness of unilateral sequential video-assisted thoracic sympathetic chain clamping (VATSCC) by clip application in children focusing mainly on the incidence of postoperative CH.

## Materials and methods

**Figure 1:**
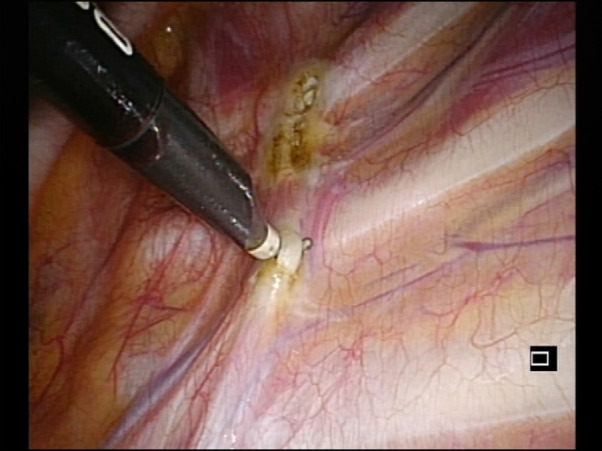
Identification of the sympathetic nerve.

**Figure 2:**
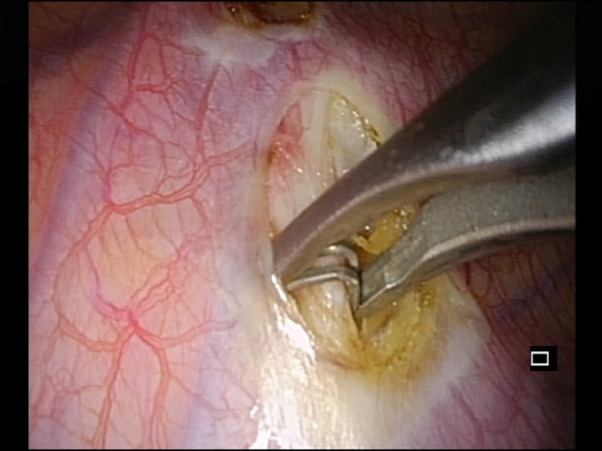
Application of the clip.

All our patients had excessive sweating of the palms and experienced excessive hyperhidrosis leading to considerable social embarrassment that interfered in a negative way with the daily activities. The most consistent problems were embarrassment on shaking hands, difficulties in writing and drawing and difficulties relating with the opposite gender. All patients resulted unresponsive to medical treatment for at least 6 months. The contralateral procedure was performed 2 months after the first one. All patients had a severe type of primary hyperhidrosis of the hands. A preoperative *x*-ray of the chest was performed on all patients to rule out any kind of anomaly. After obtaining informed consent from the parents, the surgical procedure was performed with the patients under general anesthesia using single-lumen endotracheal intubation in the semi-sitting position, with the arms extended at 90 degrees. A 5 mm camera was then inserted using an atraumatic Versaport® trocar with a stab incision at the level of the middle axillary line in the fourth intercostal space. Under direct visualization, a second 5-mm trocar was inserted in the second intercostal space always at the level of the middle axillary line. A pressure of 5 mm Hg was used in all the cases. The sympathetic chain was identified, and the posterior parietal pleura opened (Figure 1). Two clips were applied above and under the third rib, if possible under the parietal pleura Figure 2). The Kunz fibers, where present, were transected. Complete drainage of pleural air was obtained under the direct vision of the camera. After the full expansion of the lung, the ports were removed, and the port sites were sutured with absorbable stitches, without chest tube placement. A postoperative chest radiograph was performed the next day. All the patients were followed up and assessed for recurrence of sweating, changes in the amount of sweating in both hands, and the amount of compensatory sweating.

## Results

From August 2017 to September 2021, 58 patients (male:female ratio 32:26), mean age 16.5 years (range 14–19) (Table 1), with PPH underwent unilateral sequential VATSCC by positioning two clips, the first one at the level of the third and the second one, at the level of the fourth rib, starting on the dominant hand. The mean operation time was 21 ± 9 min (range 12–35). All patients except one (transient pneumothorax) were discharged on the first post-op day. No immediate or late complications have been recorded. The mean follow-up was 22 months (range 6–41) (Table 1). All patients except one (1.7%), affected by Raynaud's disease, showed a complete resolution of the symptoms. Seven patients (12%) developed transient moderate compensative hyperhidrosis. Five out of 7 patients developed CH at the level of upper limb while the last 2 patients showed CH at the level of lower back. In all these cases CH developed immediately after the first procedure. All these patients manifested a complete and spontaneous resolution of CH in 9 ± 3 weeks after the second surgery. In one case, the surgical procedure performed on the dominant hand (right side), resulted in the resolution of the symptoms also in the contralateral side.

**Table 1:** Characteristics of patients.

Characteristics of patients	
Age (years)	16.5
Sex (male:female)	32:26
Operation time (mean ± SD)	23 ± 6 min
N° of patients	58
N° of procedures	115
Follow-up (Mean-Range)	22 (6–41) months

## Discussion

Children with severe PPH have profuse amounts of sweat on their hands. Schoolwork, sports, social interactions, and normal daily activities may become embarrassing and very uncomfortable (6). Usually, patients affected by PPH have anxiety and often avoid social situations. An effective and safe treatment to remove this obstacle becomes desirable to improve social life. Several conservative treatments have been proposed in the last years but, their effectiveness is far from perfection (7, 8). Aluminum chloride-based antiperspirants are a well-established first-line option for all types of PPH (9). The mechanism of action is *via* aluminum salt blockade of the eccrine sweat gland ducts but, in many cases, the sweat gland function returns to normal within one week and further treatments are needed to maintain sweat control (10).

Iontophoresis involves an electric current application to enhance the transdermal delivery of an ionized substance, i.e., water, through intact skin immersed in liquid (11–13). Primarily restricted to palmoplantar locations, this modality can be utilized as an effective first or second-line option, usually after the failure of antiperspirant agents (11). This procedure needs to be performed at least 3–4 times per week to obtain an appreciable result (14). Iontophoresis is considered a safe procedure however, side effects such as burning or tingling sensations are reported by most patients.

Botulinum Toxin A injection (BTAI) is considered another viable conservative option to treat PPH. Although response rates seem to be high (80%–90%), the anhidrotic effect manifests usually three days after injections and can be maintained for about 6–9 months. For long-term successful therapy, the injections must be repeated regularly. Possible side effects include injection-site pain, discomfort, and/or local irritation (15).

**Systemic therapies such as Anticholinergic agents, Glycopyrrolate, and Methantheline bromide have been proposed with appreciable results even if most patients that do not want to continue with this medication because of its secondary effects** (16–18).

In our department, VATS represents the last step in all cases refractory to conservative treatments. This procedure involves video-assisted thoracoscopy to interrupt the sympathetic chain by transecting, resecting, ablating, or clamping the involved T3 or T4 ganglion. The principal criteria of inclusion are age <20 years, early disease onset, body mass index (BMI) <28 kg/m2, absence of both nocturnal sweating, and bradycardia and absence of significant comorbidities (19, 20). The first thoracoscopic treatment was described by Kux in 1954 (21), with excellent results in 55 patients. Video-assisted thoracoscopy sympathectomy/sympathotomy involves different and various techniques with changes in the number of ports and methods of sympathetic chain interruption. Several authors reported their experience using a single port leading to less post-operative chest pain, but no statistically significant differences have been reported in terms of postoperative pain between single port and multiport (20). Although VATS represents a permanent cure for PPH, CH often may complicate the procedure (22, 23).

Compensatory hyperhidrosis is a postoperative, often irreversible condition of excessive sweating in other anatomical districts, mainly the back, legs, abdomen and gluteal region (24). The severity of CH has been reported to be lower in patients treated for PPH compared to those treated for craniofacial or axillary hyperhidrosis (23). However, this side effect may be worse than the original problem, influencing the postsurgical quality of life (25). Compensative hyperhidrosis may occur in 3%–90% of patients who have undergone the simultaneous bilateral while the incidence decreases to 12.2% in those patients undergoing the sequential VATS with no patient suffering severe CH (24). Unilateral sequential sympathectomy/sympathotomy results in a better post-operative control of CH, due to the alteration of the quantitative distribution of thermoregulatory sweating in response following the unilateral section of the vagal trunk (19, 26). In our case, sequential VATCC seems to be very effective in reducing the incidence of CH. For this reason, we prefer, in pediatric and adolescent patients, to perform unilateral sequential VATCC. In our experience, the clamping of the sympathetic chain starting on the dominant hand, despite needing 2 procedures, results in a lower post-operative CH rate 12%. The incidence of CH is often related to the extensiveness of the sympathectomy (26). According to our experience, sympathetic chain blockage by clipping the thoracic sympathetic trunk, results in a reduction of post-operative CH, due to the absence of sympathectomy even if this procedure is not reversible (24). In all these cases except one affected by Raynaud disease, the CH disappeared spontaneously a few days after the procedure and, in one case (1.7%), a single procedure on the dominant hand was effective to resolve the problem bilaterally.

## Conclusions

Unilateral sequential VATCC with application of clips on the vagal trunk, at the level of T3–T4, may be considered, as a safe, effective and definitive treatment achieving the complete dryness and reducing compensatory hyperhidrosis in pediatric age.

## Data Availability

The raw data supporting the conclusions of this article will be made available by the authors, without undue reservation.
